# Implementation of COVID-19 vaccination services in prison in six European countries: translating emergency intervention into routine life-course vaccination

**DOI:** 10.1186/s12889-024-18063-2

**Published:** 2024-04-10

**Authors:** Sara Mazzilli, Nicola Cocco, Davide Petri, Babak Moazen, Alicia Rosello, Jemima D’Arcy, Emma Plugge, Laura Baglietto, Eva Murauer, Heino Stöver, Tassos Trattonikolas, Iakovos Stylianou, Svetlana Doltu, Vladislav Busmachiu, Josefina Mavrou, Ioanna Yiasemi, Irina Barbiros, Filipa Alves da Costa, Fadi Meroueh, Roberto Ranieri, Lara Tavoschi

**Affiliations:** 1 Department of Translational Research and New Technologies in Medicine and Surgery, University of Pisa, Pisa, Italy; 2 Scuola Normale Superiore, Pisa, Italy; 3 Infectious Diseases Service, Penitentiary Health System, Azienda Socio-Sanitaria Territoriale Santi Paolo e Carlo, Milan, Italy; 4 Department of Health and Social Work, Institute of Addiction Research (ISFF), Frankfurt University of Applied Sciences, Frankfurt/Main, Germany; 5 Heidelberg Institute of Global Health, Heidelberg University, Heidelberg, Federal Republic of Germany; 6 UK Health Security Agency, London, UK; 7 Primary Care, Population Sciences and Medical Education, Faculty of Medicine, University of Southampton, Southampton, UK; 8 Department of Clinical and Experimental Medicine, University of Pisa, Pisa, Italy; 9 Health Unit of the Villeneuve-les-Maguelone prison, University Hospital Centre Montpellier, Montpellier, France; 10 Ministry of Justice and Public Order - Cyprus Prison Department, Nicosia, Cyprus; 11 Act For Involvement, Chișinău, Republic of Moldova; 12 National Administration of Penitentiaries, Chișinău, Republic of Moldova; 13 Cyprus National Addictions Authority, Nicosia, Cyprus; 14 Health in Prisons Programme, WHO Regional Office for Europe, Copenhagen, Denmark

**Keywords:** Prison, Vaccination, COVID-19, Europe, Health inequities

## Abstract

**Background:**

Evidence has shown that the risk of transmission of SARS-CoV-2 is much higher in prisons than in the community. The release of the COVID-19 vaccine and the recommendation by WHO to include prisons among priority settings have led to the inclusion of prisons in national COVID-19 vaccination strategies. Evidence on prison health and healthcare services provision is limited and often focuses on a single country or institution due to the multiple challenges of conducting research in prison settings. The present study was done in the framework of the EU-founded project RISE-Vac. It aimed to analyse the best practices and challenges applied in implementing COVID-19 universal vaccination services during the pandemic to support future expansion of routine life course vaccination services for people living in prison (PLP).

**Methods:**

Two online cross-sectional surveys were designed and piloted: survey1 on prison characteristics and (non-COVID-19) immunisation practices; survey2 on the implementation and coverage of COVID-19 vaccination with open-ended questions for thematic analysis. Each RISE-Vac project partner distributed the questionnaire to one or two prisons in their country. Answers were collected from eight European prisons’ directors or medical directors between November 2021-May 2022.

**Results:**

According to our findings, the implementation modalities of COVID-19 vaccination services in the surveyed prisons were effective in improving PLP vaccination coverage. Strategies for optimal management of the vaccination campaign included: periodic time slot for PLP vaccination; new staff recruitment and task shifting; distribution of informational material both to PLP and prison staff. Key challenges included continuity of care after release, immunisation information system, and vaccine hesitancy.

**Conclusions:**

To the best of our knowledge, this is the first study describing the implementation of COVID-19 vaccination services in European prisons, suggesting that the expansion of vaccination provision in prison is possible. There is no unique solution that will fit every country but commonalities likely to be important in the design and implementation of future vaccination campaigns targeting PLP emerged. Increased availability of vaccination services in prison is not only possible, but feasible and highly desirable, and can contribute to the reduction of health inequalities.

**Supplementary Information:**

The online version contains supplementary material available at 10.1186/s12889-024-18063-2.

## Background

According to most recent data, more than 1,4 million individuals are detained on any given day in the European region [1]. However, due to the high turnover, the number of people who navigate through European prisons each year is considerably higher. Individuals who experience incarceration often belong to socially marginalised and disadvantaged population groups. Regardless of the limited availability of data and differences between countries, there is overwhelming evidence that people living in prison (PLP) disproportionately experience complex, co-occurring health problems, including non-communicable, infectious diseases, mental illness, cognitive disability and substance dependence [2, 3].

Despite the greater health needs experienced by PLP, these individuals often have suboptimal access to healthcare services in the community, including effective preventive services such as vaccination [4, 5]. The available evidence, albeit scarce, indicates that individuals who enter the prison system are under-immunised, particularly against Hepatitis B Virus (HBV), influenza, measles mumps and rubella (MMR), and pneumococci [6]. However, the availability of vaccination services in European prisons is limited and usually focused on a few vaccines, such as HBV [7, 8]. Routine data on vaccination coverage at entry and on vaccination uptake during incarceration episodes are largely unavailable, with direct implications on health needs, impact of services and for health planning purposes [6, 8].

Furthermore, the provision of health services in prison is heterogeneous across Europe, more frequently under the responsibility of the Ministry of Justice (MoJ) alone or with shared responsibilities with the Ministry of Health (MoH). The diverse set-ups may have implications on the availability or organisation of infrastructures, human resources and medical commodities [3, 9]. The COVID-19 emergency has uncovered some of the above-mentioned challenges, including within-country variability in policy measures adopted, highlighting important shortcomings of prison health provision and planning across the region [10, 11].

Evidence has shown that the risk of transmission of SARS-CoV-2 is much higher in prisons and other closed settings [10]. Multiple large outbreaks of COVID-19 have been documented in detention facilities worldwide [11, 12]. While non-pharmacological preventive measures have been implemented virtually in all prisons in Europe, the extent and continuity varied substantially across space and time [10, 12]. The release of the COVID-19 vaccine in late 2020 and the recommendation by WHO [13] to include prisons among priority settings have led to the inclusion of prisons in the national COVID-19 vaccination strategy [13, 14]. The implementation of universal vaccination services in prison settings has been unprecedented and such experience may provide solid ground for the much needed future expansion of vaccination offers for PLP [15]. In 2021 the project “RISE-Vac - Reaching the hard- to-reach: Increasing access and vaccine uptake among the prison population in Europe”, co-funded by the European Union, was launched to explore ways to promote vaccine offer and uptake in prisons in Europe, involving six countries across the region (Supplementary material, Table 1. List of RISE-Vac partner institutions). RISE-Vac partners are in the consortium on the basis of their willingness to participate in the project and the availability of at least one prison in the country to carry out the research activities. With the present study, we aimed to analyse the best practices and challenges applied in implementing COVID-19 universal vaccination services during the pandemic in order to support future expansion of routine life course vaccination services for PLP.

## Methods

Two online cross-sectional surveys were developed by researchers (SM, DP) using Survey Monkey (https://it.surveymonkey.com) and reviewed by experts participating in the RISE-Vac project. The surveys were piloted in the RISE-Vac project partner prisons and subsequently revised. Final surveys are available in the Supplemental material (Annex1, Annex2).

The first survey focused on prison characteristics (type of prison, prison population, capacity, etc.) and (non-COVID-19) immunisation practices for PLP. It included 10 categorical and open-ended questions. The second survey was developed to gather information on the implementation and coverage of COVID-19 vaccination in prison. The survey consisted of 20 categorical and open-ended questions covering: (i) COVID-19 vaccination service set-up; (ii) service implementation and assessment; (iii) barriers; (iv) COVID-19 vaccination coverage.

The six countries participating in the RISE-Vac project were included in this study: Cyprus, France, Germany, Italy, Republic of Moldova, the United Kingdom (UK). Each RISE-Vac project partner distributed the questionnaire to one or more prisons in their country. All participating prisons were selected using a convenience sampling method. Each RISE-Vac project partner distributed the questionnaire to one or more prisons in their country. All participating prisons were selected using a convenience sampling method. To investigate possible specific features or tailored arrangements in the implementation of COVID-19 vaccination services, one female prison and one therapeutic prison (organised around a group therapy programme, where PLPs participate in daily group meetings and activities) were included. In the countries where these prisons were selected (Germany for the female prison and UK for the therapeutic prison), another prison was also included.

In the eight participating prisons the online surveys were shared by email with the prison director (survey 1) or medical director (survey 2) of the prison health unit. Answers were collected from November 2021 through May 2022 for survey 1, and from February 2022 until 31 May 2022 for survey 2.

### General population data sources

COVID-19 vaccination coverage among citizens over the age of 18 was obtained from a publicly accessible data set developed by the European Centre for Disease Prevention and Control and World Health Organization [16]. These data were available for France, Germany and Italy. COVID-19 vaccination coverage data for Cyprus, Moldova and UK were not available by age categories. The source used was: Our World in Data developed by the University of Oxford [17].

### Data analysis

The data collected through the open-ended questions have been analyzed using thematic analysis. Inductive manual coding was applied in the analysis. The data have been coded according to the following emerging categories: COVID-19 vaccination set-up; COVID-19 vaccine implementation; barriers and facilitators; COVID-19 vaccination coverage. The preliminary categories and analysis were shared with RISE-Vac partners and agreed through a consensus building approach. The results have been presented using descriptive statistics, tables, and figures. We compared the vaccination coverage of PLP with the vaccination coverage in the general population.

## Results

### Characteristics of included prison institutions

Eight prisons answered the questionnaires. The surveyed prisons’ general characteristics are presented in Table 1. Six institutions hosted pre-trial and short-term sentences PLP. Three of these also hosted long-term sentenced PLP. Four prisons hosted juvenile PLP. Three hosted only males, one only females, while the rest hosted both. One institution was a therapeutic prison. The prisons in Cyprus, France and Italy reported overcrowding.

**Table 1:** Surveyed prisons general characteristics

Country - *N* of prison in the country	Institution responsible for prison health	Prison type	Prison population sex	Capacity, *N*	Number of PLP (Occupancy level)	Average percentage of foreigners PLP	Immunization status assessment for PLP
Cyprus − 1 prison	Ministry of Health	Pre-trial, Short-term, Long-term, Juvenile	Both	543	819 (151%)	52%	Yes, verified through medical record
France - 188 prisons	Ministry of Health	Pre-trial, Short-term, Juvenile	Male	590	960 (163%)	21%	Yes, patient history taking
Germany − 179 prisons	Ministry of Justice	Pre-trial, Short-term, Long-term, Juvenile (prison 1)	Female	300	250 (83%)	12%	Yes, patient history taking
		Pre-trial, Short-term, Long-term, Juvenile (prison 2)	Both	1400	1350 (96%)	60%	No
Italy − 206 prisons	Ministry of Health	Pre-trial, Short-term	Both	695	843 (121%)	50%	Yes, verified through medical record
The Republic of Moldova* − 17 prisons	Ministry of Justice	Pre-trial, Short-term	Both	1200	811 (68%)	4%	Yes, verified through medical record
The UK − 141 prisons	Ministry of Health (prison 1)	Open, resettlement	Male	335	309** (92%)	4%	Some verified through medical records, patient history taking
	(prison 2)	Closed, therapeutic	Male	238	163** (69%)	6%	Some verified through medical records, patient history taking

*Countries where vaccination services were not available before the COVID-19 emergency

**Prison 2 operating under lower occupancy during fire safety works, Prison 1 returning to maximum operational capacity following work to infrastructure

Vaccines for PLP were provided by the MoH for the prisons located in Cyprus, France, Italy, and UK, while the MoJ were responsible for vaccine provision in the German and Moldovan prisons. Immunisation status was always assessed upon entry into prison with the exception of one prison in Germany. In the Cypriot, Italian and Moldovan prisons the immunisation history was verified via medical records, in British prisons this is only true for part of the PLP. The alternative mode was patient history taking, also used in French and German prisons.

Figure 1 of the supplementary materials shows the vaccinations offered by each prison in the study. At the time of the study, Moldova had no active vaccination prevention service.

### COVID-19 vaccination set-up

The answers to the questionnaire show that all six countries had a strategy for COVID-19 vaccination targeting PLP. Prison universal vaccination started in March 2021 in all prisons except prison 2 in Germany and the British prisons, where it started in December 2021. Responsibility for organising the vaccination campaign was assigned to the prison health unit in five prisons (France, Germany, Italy and UK), and to the national prison administration in two prisons (Cyprus and Moldova).

Six prisons (Cyprus, Germany, Italy, Moldova, and UK) reported individual protection and avoiding cases of serious illness as an objective of the prison vaccination campaign. Three prisons (France, Italy and UK) reported the achievement of herd immunity as one of the objectives, and two prisons (Moldova and UK) mentioned also ensuring access to vaccination for all, reducing health inequities.

### COVID-19 vaccine implementation

**Fig. 1:**
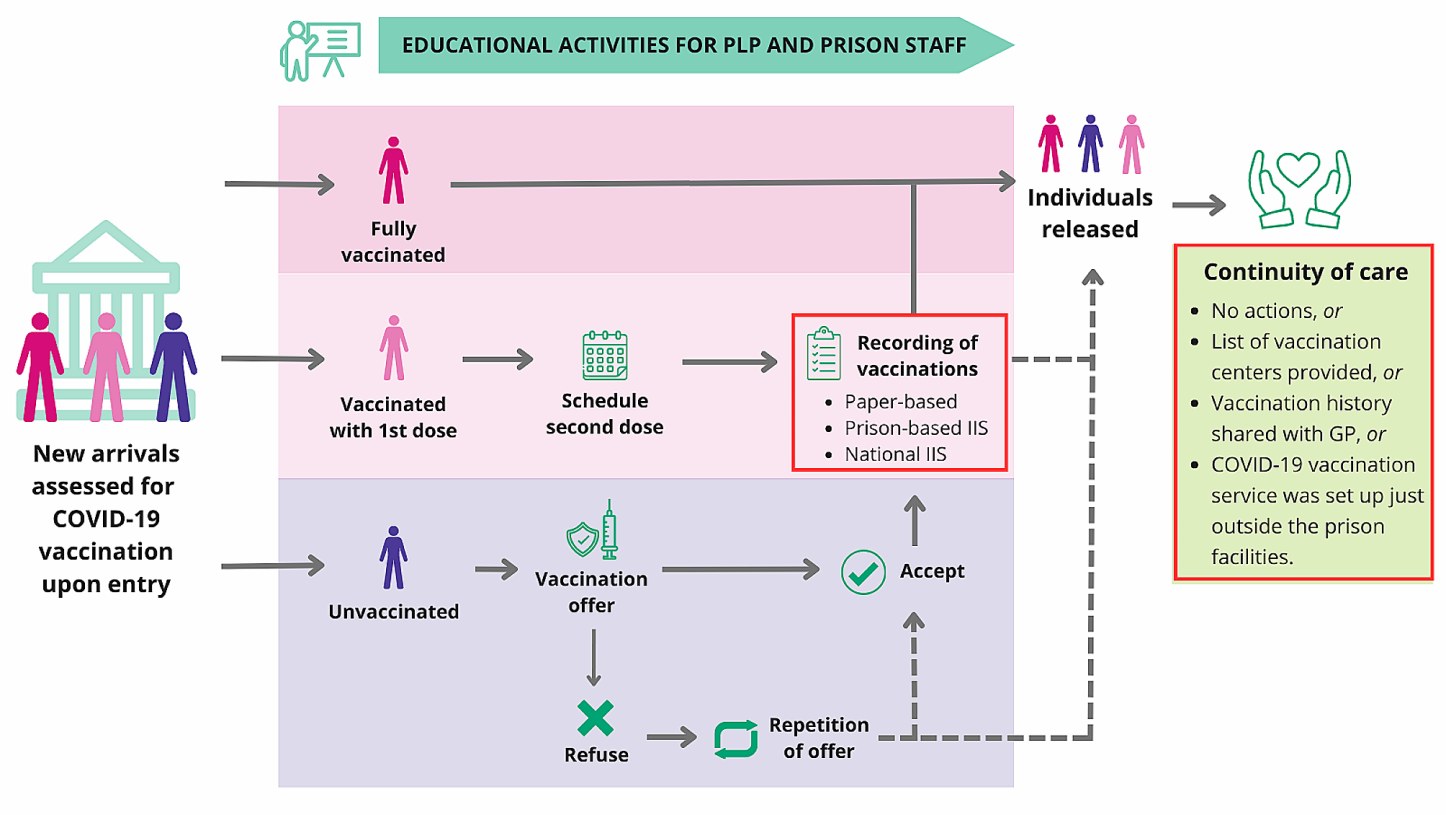
COVID-19 vaccination pathway implemented in European prisons in the study

*Red boxes in the vaccination pathway correspond to steps of the COVID-19 vaccination services implemented differently in different prisons.*

The COVID-19 vaccination pathway is summarised in Fig. 1. In all prisons involved in the survey, COVID-19 vaccine immunisation status was assessed at prison entry (Table 1). The proportion of new arrivals in prison who received at least one dose of the COVID-19 vaccine before imprisonment ranged from 30% in France and in one prison in Germany to 90% in in Italy. In the French prison, individuals not immunised were offered the vaccine immediately at the first visit. Offer was repeated to those who refused. In all other prisons, administration of the vaccine was scheduled at the next available opportunity for individuals not immunised at entry. In the prisons in Cyprus, Italy, Germany and UK, regular vaccination clinics (i.e. once a week) were organised. In six institutions (Cyprus, France, Italy, Moldova, and UK) vaccination was administered by prison healthcare staff, whereas in the German prisons it was administered by external healthcare providers for the first six months of the vaccination campaign after which the prison health unit took over. To implement the vaccination campaign, additional human resources were hired in the Italian prison and task shifting occurred in the Italian and Moldovan institutions. In these prisons, the nurses held managerial and organisational roles and the custodial staff participated in the recall of PLP who needed to be vaccinated. Despite these differences, in all the institutions, mass COVID-19 vaccination took place within prison facilities and included education/training activities tailored for prison staff (Custodial and healthcare staff) and PLP. The educational activities implemented for health personnel were: face-to-face meetings, online information sessions and leaflet distribution. Regarding changes in the supply chain for the COVID-19 vaccine, the British prisons reported a simplification of the procedures to order vaccine doses, while the Italian prison reported a faster and more flexible delivery. In the Cyprus prison vaccinations administered were recorded in paper-based prison vaccination registries, in UK institutions in a prison-based digital information system and in the Moldovan and French prisons in the national digital information system. In the Italian prison, registration of vaccinations was initially paper-based, but the possibility of adding the information to the national digital information system was later introduced (Table 2).

**Table 2:** Features of COVID-19 vaccination models of care in prisons of selected European countries

	Covid-19 vaccine campaign implementation modalities	Country of implementing prison
COVID-19 vaccination assessment and offer	COVID-19 vaccination assessment at prison entry	All
	Vaccine administered immediately at the first visit	France
	Vaccine scheduled at the next available opportunity	Cyprus, Germany, Italy, the Republic of Moldova, the UK
Vaccination supply chain	Simplification of the procedures to order vaccine doses	The UK
	Faster and more flexible delivery	Italy
Vaccine administration	Organization of regular vaccination clinics	Cyprus, Italy, Germany, the UK
	Took place within prison facilities	All
	Prison healthcare staff administering COVID-19 vaccination	Cyprus, France, Italy, the Republic of Moldova, the UK
	External healthcare providers administering COVID-19 vaccination	Germany (for the first 6 months)
	Recruitment of additional human resources needed	Italy
	Staff shifting applied	Italy, the Republic of Moldova
Educational training	Dedicated materials and information sessions for PLP	All
	Dedicated materials and training sessions For prison staff (Custodial and healthcare staff)	All
COVID-19 immunisation information system	Paper-based prison vaccination registries	Cyprus, Italy*
	Prison-based digital information system	The UK
	National digital information system	France, the Republic of Moldova, Italy
Continuity of care	List of vaccination centres provided to PLP upon release	France, the UK
	Vaccination history transferred to community-based GP (for individuals resident in the country of detention)	The UK
	COVID-19 vaccination service for released individuals within prison premises	Italy
Vaccination campaign evaluation	Monitoring and evaluation framework applied	The UK

**Fig. 2:**
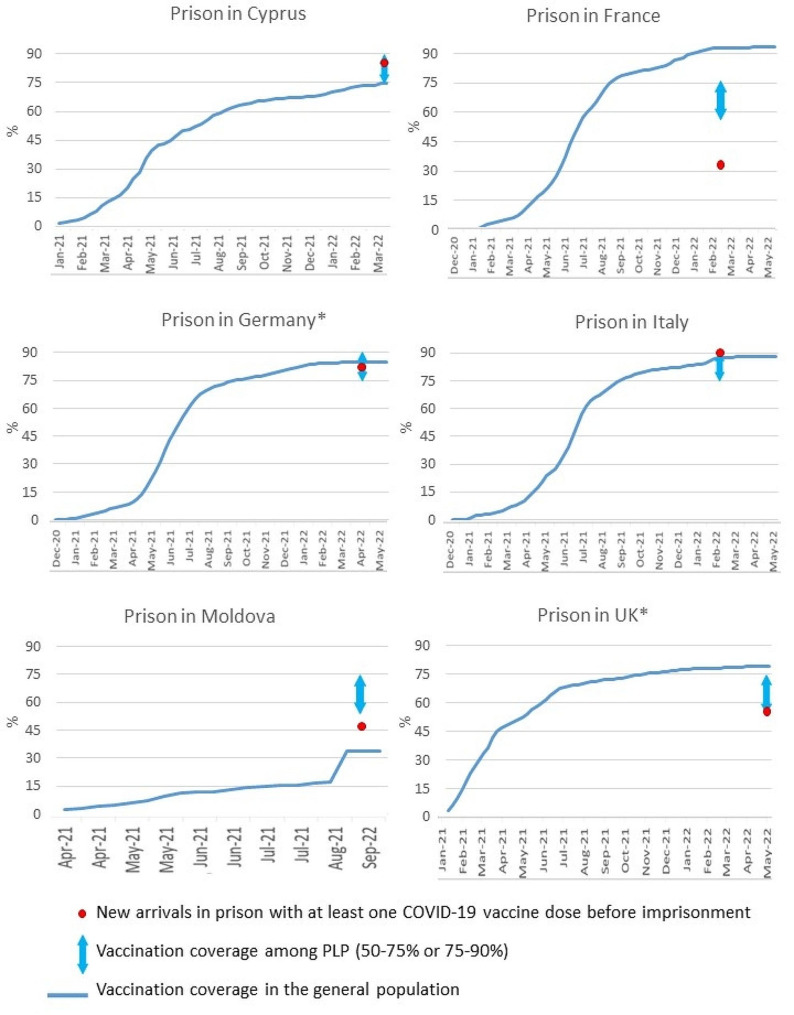
Vaccination coverage among new arrivals, PLP in the prisons surveyed and the general population (Feb-May 2022). *For Germany the data are shown for the prison with PLP of both sexes, for UK data from the non-therapeutic prison are shown

In the French prison, PLP were given a list of vaccination centres where they could receive booster doses in the community. In the British prisons surveyed, in addition to providing the list of vaccination centres in the community, the vaccination history of the released individual was transferred to community-based GP (for individuals resident in the country of detention), while a COVID-19 vaccination service for released individuals was set-up within the Italian prison premises. There was no protocol in the Cypriot and Moldovan prisons to link to care subjects who were released before completing the COVID-19 vaccination schedule. The evaluation of the vaccination campaign was carried out by the Italian and British prisons. Continuous assessment of vaccination uptake rates among PLP was regularly performed and the findings were used to inform corrective actions such as repeated offers to all eligible individuals and review of information/communication material. At the time of the survey, the vaccination uptake of the complete series of COVID-19 vaccine among PLP in prison was between 50 and 75% in the France Moldovan and British prisons, and between 75 and 90% in Cypriot, German and Italian prisons (Fig. 2).

### Barriers and facilitators

Common barriers encountered during the upscale of COVID-19 vaccination services were reported by all respondents. Identified barriers related mostly to infrastructural constraints, such as lack of human resources to deal with the surge of activity and lack of commodities such as adequate cold supply chain systems. These were addressed in different manners including task shifting, with lower cadres managing patients’ engagement and appointments, or implementation of pragmatic solutions such as dedicated immunisation days (Table 3).

**Table 3:** Reported barriers and solutions implemented during COVID-19 universal vaccination campaigns in selected European prisons

Reported challenges	Identified solutions	Actions for sustainability
Shortage of health staff	Task shifting (engagement of nurses, social workers and custodial staff)	Revise and adapt the allocation of tasks within prison HCWs and prison staff to promote a “whole-of-prison” approach to healthcare services and health promotion
	Additional staff hired	Collective vaccine administration was implemented for other vaccinations to reduce staff requirement
Challenges of cold supply chain	Designated vaccination days for collective immunisation of PLP	Collective vaccine administration implemented for other vaccinations
	Easier vaccines ordering and more flexible deliveries	
Paper-based vaccination registries not inter-operable with community IIS	Access to community IIS enabled for prison healthcare services - access restricted to COVID-19 vaccine information	Ensure interoperability between prison and community IIS. Ensure interoperability between prison and community health record
Lack of referral system post-release	Set up of dedicated service for released individuals within prison premises	Establish procedures to plan appointments in the community vaccination centres (to benefit from interoperability of prison and community IIS)
	Provision of a list of community services where to receive COVID-19 vaccine booster doses	
High level of vaccine hesitancy	The organisation of information sessions tailored to PLP and HCWs/prison staff; Development of tailored information material for PLP and HCWs/prison staff	Planning regular educational and information activities on vaccination targeted towards PLP and HCWs/prison staff; to be combined with regular educational and information activities on other relevant health issues

Other important barriers related to recording the administration of vaccine doses in the absence of an interoperable immunisation information system between prison and community, or to the use of paper-based records exclusively. While this was successfully addressed in some cases (e.g. Italian prison) during the COVID-19 emergency, the solutions achieved were restricted to COVID-19 immunisation.

The post-release referral was also recognised as a barrier to the completion of the vaccination course. Solutions identified were common to other conditions requiring continuous access to care services (e.g. HIV treatment, opioid agonist treatment) [22] and included setting appointments in the most convenient healthcare service in the community (active referral) or providing the individual with a list of relevant services (passive referral). In one case (Italian prison), an ad hoc COVID-19 vaccination service accessible to released individuals was set up within prison premises. Released individuals were recalled and given appointments for booster dose/s. Low vaccination uptake has been reported as a limitation for achieving high vaccination coverage in PLP. The use of information material and the organisation of educational activities concerning vaccinations have been reported as facilitators to decrease vaccination hesitancy. All respondents reported that the implementation of COVID-19 vaccination services in prison was likely to impact future vaccination services for this population. In particular, respondents underlined the importance of sustaining and translating the good practices introduced with COVID-19 vaccination to other immunisation programmes relevant to PLP.

## Discussion

To the best of our knowledge, this is the first study describing the implementation of COVID-19 vaccination services in European prisons, showing that the expansion of vaccination provision in prison is possible, regardless of whether the health of PLP is the responsibility of the MoH or the MoJ. There is no unique solution that will fit every country’s prisons although these findings could inform the design and implementation of future vaccination campaigns targeting PLP.

One key element was the organization of a periodic (i.e. one day per week) time slot for PLP vaccination. This reduced the workload of prison healthcare staff compared to ad-hoc vaccination. Staff could accomplish vaccination-related activities in defined time slots, allowing more time for routine healthcare activities. This also addressed possible cold supply chain issues by streamlining vaccination delivery, stocking and administration in a single day. It is also likely to have decreased vaccine wastage [15].

With the exception of German prisons, respondents in the other five countries reported the exclusive involvement of healthcare staff working in prisons for COVID-19 vaccination of PLP. Despite this, all prisons recognised the need and implemented specific training activities tailored to healthcare professionals to equip them with the relevant skills for the administration of vaccines to PLP. This is in line with evidence from the literature related to both healthcare workers who administer vaccinations in other settings [18, 19] and health workers who work in prison but address other health issues [20, 21].

In response to staff shortages, task-shifting was implemented in two prisons involving other professionals for health and custodial staff. As already highlighted at global level for other primary health interventions and prevention tasks [22, 23], this represents a useful organisational approach to improve coverage and timeliness of health prevention activities in limited resource settings.

Finally, high vaccination hesitancy was reported among PLP in several prisons, leading to low vaccination acceptance in some instances. Vaccine hesitancy is one of the most important reported barriers towards controlling vaccine-preventable diseases in prisons [24]. PLP may refuse vaccination due to various reasons including but not limited to concerns about side effects, low levels of perceived risk, distrust of authorities, vaccine, or vaccinator, or even fear of needles and injections [24, 25]. To address this issue, in all responding prisons before or in parallel with the introduction of COVID-19 vaccination, efforts were made towards the development and dissemination of tailored information and educational resources.

There were different levels of information technology implementation within the prisons included in the study. Paper-based IIS was used in some cases as the main recording system. Electronic records were in use at most prisons, however interoperability with the national health information system was not always available. This is in line with what is reported in other prison institutions in Europe [26].

Continuity of care and completion of the vaccination cycle after release was identified as an issue in all prisons included. An ad hoc COVID-19 vaccination service, accessible to released persons, was set up within the Italian prison premises. Although effective in addressing pandemic emergency needs, this strategy is hardly sustainable. In the British prisons surveyed, vaccination history of released individuals was shared with the assigned community general practitioner where possible. Efforts to guarantee adequate continuity of care can be severely hampered if individuals’ medical records cannot reach healthcare services in the community upon release, or prison health services upon admission. Lack of accurate, updated and accessible data on previous immunisation, or other health interventions, may result in inadequate or untimely actions. This lack of seamless transfer of electronic information has been well documented for many other health conditions in people leaving prison in a number of countries [27].

Improvement in the prison-based health data collection is also pivotal to support the development of evidence-based approaches. Only two of the six prisons included in the questionnaire reported having a formal system to evaluate the effectiveness of the vaccination campaign.

Therefore, while the COVID-19 pandemic has catalysed some progress, improving the prison health information systems remain a major area for future investment. Ensuring interoperability of individuals’ and population (aggregated) health information system between prison and community is an essential requisite to guarantee continuity of care for people transitioning through the penal system, as well as to allow healthcare monitoring and planning activities to be performed at the same standard as in the community and using a data-driven approach, ultimately promoting health equity [5].

According to our findings, the implementation of COVID-19 vaccination in prison was effective in improving PLP vaccination coverage, including in countries such as Moldova, where vaccination services in prison were not available before the COVID-19 pandemic. Interestingly, this was achieved in prisons across countries with different governance arrangements with respect to prison healthcare provision [28]. There are various factors that may have impacted on the increase in coverage observed that are beyond the scope of this study but should nonetheless be mentioned. Namely, the adoption of national vaccination plans that directly refer to PLP, the mere acceptance that there is equivalence of care and thus PLP are included or, in contrast, the explicit exclusion of prisons from national policies. Coverage data presented in this manuscript is aligned with the scarce information available, namely from WHO, where in October 2021, Moldova reported having 36.8% of PLP fully vaccinated against COVID-19 [14]. Similarly, UK reported a higher vaccination coverage by June 2021 (66%). The speed of uptake in these two particular cases may be well related to the vaccination policies adopted, respectively omitting prison populations or explicitly acknowledging them as a group at higher risk.

This study has several limitations. First, the selection of prisons is convenience based. Second, the number of prisons interviewed is limited. Therefore, the results presented may not be transferable to all prisons in the countries studied. To increase representativeness the investigation of effective immunization programs implemented in prisons should be extended in future studies to a larger sample.

## Conclusion

While this study captures the experience of only a few prison institutions within selected countries and a single vaccine, the COVID-19 emergency has shown the potential to exploit incarceration as a time window and prison as a place to offer targeted immunisation programs. This is particularly relevant, as our findings showed that availability of vaccines, beyond COVID-19, is limited in the surveyed prisons, confirming existing evidence, despite international guidelines extensively recommending vaccination for PLP [6, 13].

Providing expanded, age-appropriate, free from coercion and tailored vaccination services to PLP will contribute to improving the health status of individuals in detention, while reducing within-prison transmission. Moreover, PLP transition to the community and therefore the health benefits resulting from vaccination accrue into the community [4, 29]. The potential utility and desirability of expanded vaccination programmes in prison settings is even more evident when framed within regional and global initiatives such as the elimination of measles and rubella [30], the efforts to eliminate vaccine preventable cancer [31] and the achievement of universal health coverage, including access to safe, effective, quality and affordable vaccines for all is a component of UN Sustainable Development Goal 3.8 [32].

Life-course vaccination in prison should be part of an integrated healthcare system aimed at preventing and mitigating the burden of infectious diseases. As reported by the WHO, providing integrated services to combat multiple infectious diseases can synergistically strengthen the response, expand coverage and alleviate stigma and discrimination [33]. The implementation of life course vaccination programmes in prisons requires full integration of prison health into public health and is essential to upholding the principle of equity of care [34] and to guarantee the right to health for those deprived of liberty, leaving no one behind.

In conclusion, the COVID-19 pandemic has further confirmed how increased availability of vaccination services in prison is not only possible, but feasible and highly desirable, and can contribute to the reduction of health inequalities.

### Electronic supplementary material

Below is the link to the electronic supplementary material.

Supplementary Material 1

## Data Availability

The data used and/or analysed during the current study are available from the corresponding author on reasonable request.

## Abbreviations

PLP: People living in prison
HBV: Hepatitis B Virus
MMR: Measles mumps and rubella
MoJ: Ministry of Justice
MoH: Ministry of Health
UK: The United Kingdom
