# Supplementary material for: Implementation of COVID-19 vaccination services in prison in six European countries: translating emergency intervention into routine life-course vaccination
Source: BMC Public Health. 2024 Apr 10;24:1001. doi: 10.1186/s12889-024-18063-2 (PMC11007954; doi:10.1186/s12889-024-18063-2)

| **Applicant Organisation Name** | **Country** |
| --- | --- |
| University of Pisa (UNIPI) | Italy |
| Frankfurt University of Applied Sciences (FRA-UAS) | Germany |
| ASST Santi Paolo e Carlo Presidio Ospedale San Carlo Borromeo - San Paolo Hospital (SPH) | Italy |
| Department of Health - Public Health England (PHE) | United Kingdom |
| National Administration of Penitentiaries (NAPM) | Moldova |
| Centre Hospitalier Universitaire Montpellier (CHUM) | France |
| Health Without Barriers (HWB) | Italy |
| Cyprus National Addictions Authority (NAAC) | Cyprus |
| Ministry of Justice and Public Order - Cyprus Prison Department (CPD) | Cyprus |

Table 1. List of RISE-Vac partner institutions

**Annex 1. Prison characteristics survey**

Section 1: General prison information

1. Prison’s country:
2. Prison type (please tick all that apply):

- pre-trial & short-term sentence
- long-term sentence
- juvenile
- general/mental hospital
- other

1. Prison population:

- Male only
- Female only
- Male and female

1. Maximum capacity of the prison:
2. Current number of detainees:
3. Average percentage of foreign national detainees within the prison population:

Section 2: Immunization practice for PLP

1. Which Institution provides vaccines to prisons for PLP?

- National Health Department
- Regional Health Department
- Department of Justice
- Department of Home Affairs
- Local Hospital
- Other (please specify)

1. Is the Health assessment of people living in prison performed at prison entry?

- Yes
- No

1. Is the immunization status of people living in prison assessed?

- Yes and verified through medical record
- Yes, but not verified through medical record
- Yes, partially assessed
- No

1. Which vaccines are usually administered in your structure?

| **Vaccines** | 🗹 | **Everyone/Risk groups** |
| --- | --- | --- |
| Hepatitis B (HBV) |  |  |
| Hepatitis A (HAV) |  |  |
| Flu |  |  |
| Human papillomavirus (HPV) |  |  |
| Diphteria, pertussis, thetanus (DPT) |  |  |
| Measles, mumps and rubella (MMR) |  |  |
| Pneumococcal disease |  |  |
| Meningococcal disease |  |  |
| Herpes zoster |  |  |
| COVID-19 |  |  |
| Other (specify) |  |  |

**Annex2. Implementation of COVID-19 vaccination services survey**

Section 1: COVID-19 vaccination service set-up

1. Is there a vaccination plan/strategy for COVID vaccination in prisons in your country?

- Yes
- No

1. When was the COVID-19 vaccination service set up in your institution? (Month and year)
2. What is the main goal of the COVID-19 vaccination programme in your institution?

______________________________________________________________________________________________________________________________________________________________________________________________________________________________________________________

Section 2: services implementation and assessment

1. Do you assess COVID- 19 vaccination status at prison entry?

- Yes
- No

1. Please describe how the COVID-19 vaccination service is organised in your prison:

______________________________________________________________________________________________________________________________________________________________________________________________________________________________________________________

1. Who is accountable for the vaccination services in your prison?

____________________________________________________________________________________________________________________________________________________________________

1. Was additional staff hired?

- Yes
- No

1. Have there been changes in the tasks performed by health professionals to implement COVID-19 vaccination (task shifting)?

- Yes
- No

If YES, please explain how:

______________________________________________________________________________________________________________________________________________________________

1. Was the COVID-19 vaccination service housed within your prison?

- Yes
- No

1. Were/are COVID-19 education/training activities provided to healthcare staff working in prisons?

- Yes
- No

If YES, please explain how:

______________________________________________________________________________________________________________________________________________________________

1. Do you have any programs/materials to inform PLP regarding COVID-19 vaccination?

- Yes
- No

1. Data on vaccination services are captured on:

- Individual’s paper-based medical record
- Prison-based immunization information system
- Regional/national immunization information system

1. Is there any referral protocol for individuals who are released before completing the COVID-19 vaccination schedule?

- Yes
- No

If YES, briefly describe it:

______________________________________________________________________________________________________________________________________________________________

1. Has the vaccine distribution system in prison changed compared to the past?

- Yes
- No

If yes, how?

______________________________________________________________________________________________________________________________________________________________

1. Did you assess the impact of the service?

- Yes
- No

If yes, how?

______________________________________________________________________________________________________________________________________________________________

Section 3: Barriers

1. Were there any challenges to the implementation of COVID-19 vaccination in your prison?

- Budgetary constraints
- Human resources constraints
- Infrastructural constraints
- Vaccine supply
- Lack of support from prison authorities and staff
- Policy constraints
- Other challenges

1. Are there any barriers to COVID-19 vaccination services implemented in your prison?

- Yes
- No

If YES, please specify

_____________________________________________________________________________________________________________________________________________________________________________________________________________________________________________

1. In your opinion, will the implementation of COVID-19 vaccination services in your prison influence the provision of other vaccinations in future?

Section 4: COVID-19 vaccine coverage

1. What is the proportion of unvaccinated individuals at entry?

__________________________________________________________________________________

1. What is the proportion of individuals who complete the COVID-19 vaccination schedule before release?

- >90%
- 75-90%
- 50-75%
- <50%

Fig. S1. Vaccinations offered to PLP in the investigated prisons at the time of the study.


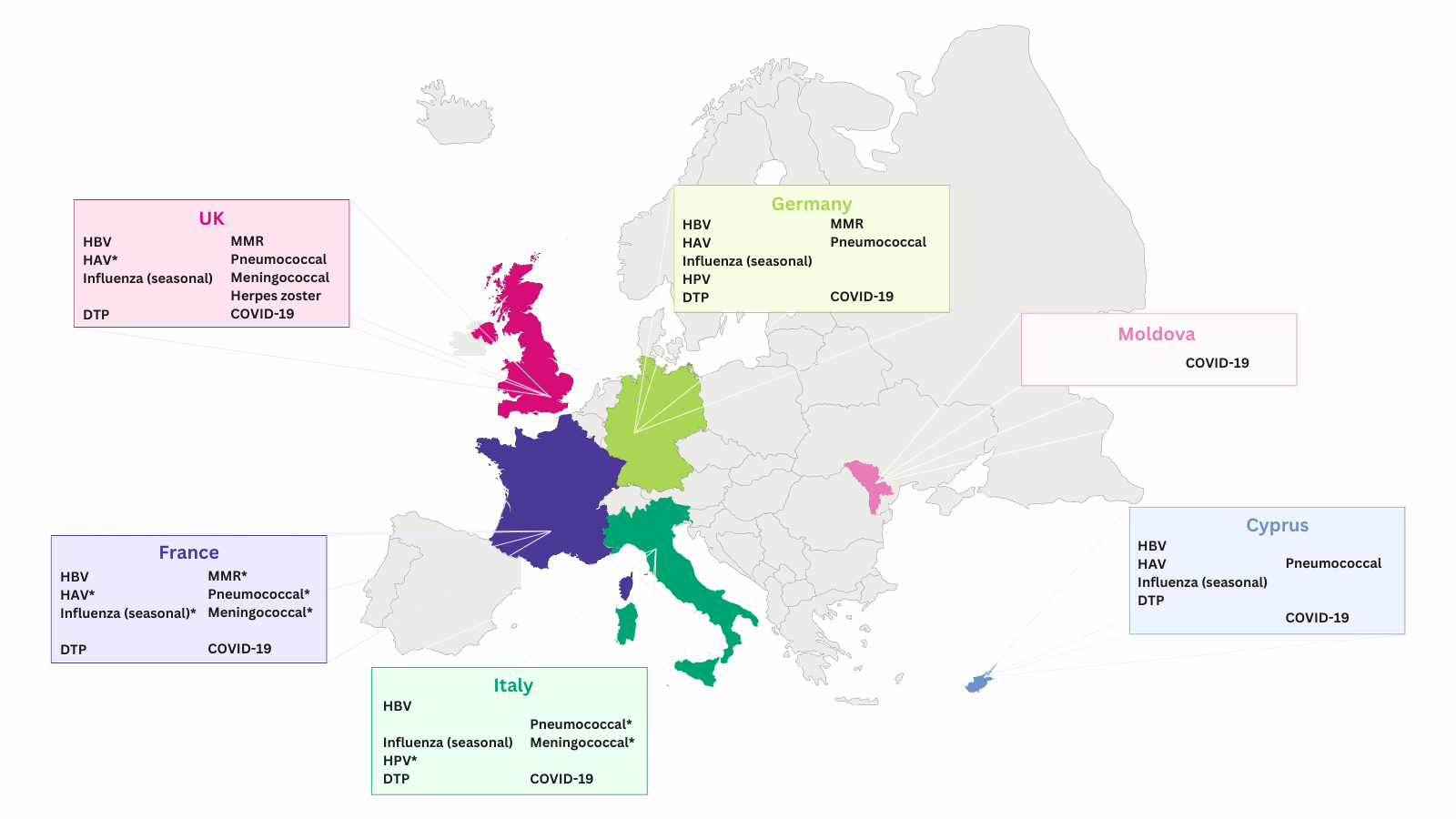

Supplement: Supplementary file 1 — Supplementary Material 1 [file 12889_2024_18063_MOESM1_ESM.docx]
